# A Fungal Sterylglucosidase at the Intersection of Virulence, Host Immunity, and Therapeutic Development

**DOI:** 10.1128/mbio.02425-22

**Published:** 2022-10-18

**Authors:** Robert A. Cramer

**Affiliations:** a Geisel School of Medicine at Dartmouth, Hanover, New Hampshire, USA

**Keywords:** *Aspergillus fumigatus*, fungal vaccine, sterylglucosidase, fungal immunity, immunocompromised, invasive aspergillosis

## Abstract

Human fungal infections (mycoses) cause significant morbidity and mortality in high-risk populations. Contemporary antifungal therapies rely heavily on three classes of antifungal drugs, and to date, no fungal vaccine is in clinical use for invasive mycosis. A major gap in knowledge related to fungal vaccine development is identifying lasting mechanisms of protective immunity in immunocompromised individuals. Recent studies in Cryptococcus neoformans and now Aspergillus fumigatus have identified a fungal sterylglucosidase essential for pathogenesis and virulence in murine models of mycoses. Fungal strains deficient in this sterylglucosidase can surprisingly also induce substantial immune-mediated protection against subsequent challenge with wild-type strains in multiple immunocompromised murine models of mycoses. Here, I discuss the implications and future directions of these exciting and impactful results.

## COMMENTARY

Invasive aspergillosis (IA), most commonly associated with the filamentous fungus Aspergillus fumigatus, is too often a lethal disease in immunocompromised individuals (1). Like most human diseases caused by fungi, epidemiological data on the true incidence and burden of IA remain limited (2). However, the treatment of IA and other human fungal diseases represents a growing and costly expenditure in health care and human life resources across the planet (3). Despite contemporary antifungal therapies, A. fumigatus and related filamentous fungi cause significant morbidity and mortality due to persistent suboptimal treatment outcomes. However, clinical and basic research advances over the last 40 years have significantly improved both the detection and treatment of IA in at-risk populations (4). In fact, the spectrum of individuals at risk for IA has changed over the last decade, in part due to medical research advances (5). While leukopenic individuals remain at high risk, individuals on steroid regimens and/or emerging new biologic therapies with qualitative immune system defects represent growing populations at risk for IA (6). Moreover, severe acute respiratory syndrome coronavirus 2 (SARS-CoV-2) and severe influenza A virus infections are now recognized as emerging and important IA risk factors (7–10). A change in at-risk populations is important from both prevention and treatment perspectives and is emphasized by prophylactic antifungal drug administration in high-risk populations, for example, in patients with acute leukemia and bone marrow transplant recipients. Prophylactic antifungal use for IA and other invasive fungal infections (IFIs) illustrates another critical point: our ability to identify individuals at risk for IFIs has significantly improved. Consequently, new opportunities exist to prevent both the initiation of an IFI and its associated morbidity and mortality.

One such opportunity is the development of fungal vaccines (11). A particular challenge with IFI vaccine development is the immune system dysfunction that is present in the most at-risk populations. For IA, hematological malignancy patients represent the most at-risk group, particularly during the preengraftment phase of an allogeneic bone marrow transplant when significant quantitative and qualitative defects in immunity are present. How does one develop an efficacious IA vaccine when the at-risk population is severely immunocompromised? To answer this question, continued basic research into the mechanisms of antifungal immunity in different clinically relevant immunocompromised settings is needed. This question seems particularly urgent as the spectrum of individuals at risk for IA continues to expand with emerging immunomodulating therapies.

In a recent study, Fernandes et al. present an exciting new tool to improve our understanding of host immunity mechanisms in immunocompromised settings relevant to IA (12). Previous research from this group identified a sterylglucosidase-encoding gene (*SGL1*) in the human-pathogenic yeast Cryptococcus neoformans that was essential for virulence in a murine model of cryptococcosis (13). The *SGL1*-null mutant was not only avirulent but also able to protect mice against subsequent lethal fungal challenges in multiple murine vaccination models (14). Inhibitors of Sgl1 were able to restrict C. neoformans infection to the lung in a murine model, suggesting that the inhibition of Sgl1 activity was also able to reduce virulence and disease progression. In the study by Fernandes et al., the authors continued their investigations into the exciting potential of targeting fungal Sgl1 homologs for therapeutic development through the generation of an *sglA*-null mutant in A. fumigatus. In two immunologically distinct murine models of invasive pulmonary aspergillosis (IPA), the *sglA*-null mutant was unable to establish invasive disease. Indeed, the avirulence of the *sglA*-null mutant is striking in these severely immunocompromised murine models. Exciting questions about the attenuated virulence of the *sglA*-null mutant remain to be explored in future studies. For example, to what extent can the *sglA*-null mutant initiate infection in these distinct immunocompromised host environments? It is fairly striking that the *sglA*-null mutant has only a modest *in vitro* growth defect yet such a profound loss of virulence. An important area for future investigation is the *in vivo* fitness of the *sglA*-null mutant in both IA models throughout the early stages of the fungus-host interaction. Intriguingly, chemical inhibition of C. neoformans Sgl1 significantly reduced growth in a low-oxygen environment, which is also present in murine models of IA (15–17).

In addition to the impressive pathogenesis attenuation of the *sglA*-null mutant, the ability of live or heat-killed *sglA* mutant conidia to protect immunocompromised mice from a subsequent wild-type A. fumigatus challenge is significant. This successful vaccination approach involved challenging the mice with *sglA* mutant conidia following the administration of the respective immunomodulating drugs, triamcinolone acetonide (a synthetic steroid) and cyclophosphamide (a cytotoxin). In this vaccine approach, the protection against subsequent fungal challenge offered by the *sglA*-null mutant in both models is quite astounding. Mechanistically, the results raise important questions about the type of immunity conferred by the *sglA* mutant strain. How does the loss of a fungal sterylglucosidase result in such dramatic protective immunity to fungal infection in immunocompromised host settings?

One possibility is the immunomodulating properties of sterylglucosides (SGs) (18). As the *sglA*-null mutant accumulated significantly more SGs in its conidia and hyphae, it is reasonable to hypothesize that SGs contribute to the observed protection through unknown mechanisms. How the immune system would be exposed to these fungal SGs warrants further study. *In vitro*, the *sglA*-null mutant displayed a significant delay in conidial germination, but the growth kinetics of the mutant *in vivo* remain to be fully studied, as discussed above. Given the extreme virulence phenotype of the null mutant, one can speculate that the mutant fails to initiate growth and subsequent infection during the vaccination phase in immunocompetent animals. Thus, the release of SGs during the early stages of infection by germinating conidia seems somewhat unlikely but cannot yet be ruled out. If mutant conidia do indeed germinate and expose/release SGs through undefined mechanisms (e.g., extracellular vesicles), their impact on immunological memory could be profound. An interesting follow-up experiment would be to vaccinate animals with heat-killed wild-type conidia that are supplemented with physiological levels of SGs to see if this strategy enhances immunity.

Alternatively, studies by those investigators clearly show that after conidial germination, *sglA*-null mutant hyphae have an altered cell surface compared to that of the parental strain. Consistent with these data, the *sglA*-null mutant displayed a significant increase in extracellular matrix production but a surprising decrease in biofilm adherence under the conditions tested. Consequently, these data suggest that the loss of *sglA* significantly impacts cell wall and extracellular matrix polysaccharide compositions through unknown mechanisms. Further studies on the cell wall composition and cell surface of the immunoprotective *sglA*-null mutant conidia are needed given the result that nonviable *sglA*-null mutant conidia were sufficient for protection in the tested IPA animal models. Can the *sglA*-null mutant adhere to host tissue during infection initiation? *In vivo* adherence quantitation is exceedingly difficult, but perhaps insights can be gained from early-infection analyses of lung architecture, fungal distribution in the lung, and fungal burdens.

Regardless of the *in vivo* adherence phenotype of the *sglA*-null mutant, it will be important to define the cell surface antigens in both the boiled and autoclaved conidia used in the vaccination protocol. An intriguing idea is that fungal SGs alone or in combination with well-known fungal pathogen-associated molecular patterns (β-glucans, for example) induce the expression of fungal pattern recognition receptors in the lung on specific cell types that remain present and functional after the initiation of immunosuppressive regimens. The use of single-cell transcriptome sequencing (RNA-Seq) approaches in the lung with the existing vaccination protocol may help reveal immune cell subpopulations required for protection and the associated molecular pathways. These are important future experiments, particularly in the context of the immunosuppressive drugs utilized in that study. Complementary to the host side approach, utilizing fungal genetics to identify fungal factors that help mediate the protective effect of *sglA* loss will be fruitful. For example, the loss of *cap59* (and, subsequently, the Cryptococcus polysaccharide capsule) in the *sglA* mutant background eliminated the protective effect of *sglA* loss (19). Would an *sglA*-null mutant in an A. fumigatus extracellular matrix-nonproducing strain still provide protection (20)? Or would mutants with altered cell wall or matrix compositions and properties provide protection? Would these mutants also possess virulence defects?

To better define the mechanism of host protection against IA, it will be important to examine the interaction of both live and dead *sglA*-null mutant conidia with the epithelia and phagocytes in the lung at the early stages of infection initiation using both the vaccination protocol and virulence models. In the vaccinated animals, do *sglA*-null mutant-vaccinated animals clear conidia faster and more efficiently than unvaccinated animals? How do boiling and autoclaving affect the interaction with the host epithelia and resident phagocytes? The fact that protection persists in the face of a neutrophil-depleting immunosuppression regimen is a surprising result. The application of the recently developed fluorescent Aspergillus reporter (FLARE) technology with a careful analysis of immune infiltrates in the lung will likely be revealing (21). Along these lines, determining what cell populations ultimately mediate this protection, and if the protection is cell mediated, will be an important research direction moving forward. Experiments in T-cell- and/or B-cell-deficient mice using this vaccination protocol will be important for uncovering the mechanism. Previous vaccination studies with crude and recombinant A. fumigatus antigens have largely pointed to CD4^+^ T-cell-mediated protection mechanisms (22). Intriguingly, *sgl1*-null mutant-mediated protection against Cryptococcus neoformans requires CD4^+^ or CD8^+^ T cells (23). However, depending on the results in T- and/or B-cell-deficient animals with the A. fumigatus *sglA*-null mutant, it may be interesting to explore the recently investigated innate immune-mediated trained immunity mechanism and models (11). Can long-lived resident phagocytes contribute to protective immunity to *sglA*-null mutant conidia in the face of clinically relevant immunosuppression regimens? Or do novel antibodies induced by *sglA*-null mutant conidia contribute to protection?

Finally, additional studies will almost certainly need to include the interaction of *sglA*-null mutant conidia with relevant human cell models. Once the mechanism(s) of protection is uncovered in mice, does this mechanism translate to the corresponding human cell populations? If the mechanism is in part T-cell mediated, as recently discovered for C. neoformans *sglA*-null mutant-mediated protection, can A. fumigatus *sglA*-null mutant conidia be used to expand donor T cells *ex vivo* for use in allogeneic hematopoietic transplant recipients? What seems clear is that further study of fungal *sglA*-null mutants is highly warranted. Now, in two different important human fungal pathogens, the loss of fungal sterylglucosidase attenuates virulence, and null mutant strains provide substantial protection against infection in murine IFI vaccination models. At a minimum, these fungal sterylglucosidase mutants should be used to better define and understand the mechanisms of fungal pathogenesis and immunity. Defining the underlying mechanisms behind the fungal and host phenotypes may yield new insights into the design of novel therapies, including vaccination, in increasingly common IFI at-risk populations. With this in mind, the current and related studies by these and other investigators provide a foundation for additional studies combining fungal genetics, pathogenesis, and immunological approaches to combat the increasing incidence and impact of human fungal infections.
